# 

**Published:** 1904-03-12

**Authors:** 


### Publications.

ENLARGED. EIGHTH EDITION. Crown 8vo. 10s. 6d.

**PHARMACY, MATERIA MEDICA, AND THERAPEUTICS**

is now rewritten and printed in large type, and is made a companion to the following volume. It contains an account of the action **of every drug in the B.P.**, as well as a section upon all the newest drugs, with an Introduction to the Art of Dispensing. With woodcuts, prescriptions, &c

Also FOURTH EDITION. 1055 pages. 8vo. 18s.

**DICTIONARY OF TREATMENT.**

Containing an up-to-date Article upon the Treatment of every Diseased Condition, whether Medical, Surgical, Gynæcological, or Ophthalmological, as well as Articles upon Poisoning, Drowning, and the various Surgical Emergencies and Accidents, arranged for Rapid Reference.

By W. WHITLA, M.A., M.D., Professor of Materia Medica, Queen's College, Belfast; Senior Physician to the Royal Victoria Hospital, &c.

The Publisher will supply both volumes, carriage paid, for 21/-.

London: HENRY RENSHAW, 356 Strand.

JUST PUBLISHED.
NEW WORK BY MR. W. ROGER WILLIAMS.

**VAGINAL TUMOURS.**

With Special Reference to Cancer and Sarcoma. 92 pp. With 4 Illustrations. Price 5s. 6d.

**DISEASES OF THE BREAST.** With Special Reference to Cancer. 21s.
**PRINCIPLES OF CANCER AND TUMOUR FORMATION.** 7s. 6d.
**INFLUENCE OF SEX IN DISEASE.** 3s. 6d.

J. BALE, SONS & DANIELSSON, Ltd., London.

Crown 8vo. Illustrated. Price 2s. 6d. net.

**ANÆSTHETICS.** By J. BLUMFELD, M.D.Cantab., Senior Anæsthetist to St. George's Hospital; Anæsthetist to the Grosvenor Hospital for Women and Children, London, and to the National Dental and Metropolitan Hospitals.

*Lancet.*—"....In writing this handbook the author has been quite successful." *Australasian Medical Gazette.*—"We can strongly commend this book." *Medical Chronicle.*—"The book is one which we can heartily recommend to senior students and practitioners."

London: BAILLIÈRE, TINDALL & COX, 8 Henrietta St., Covent Garden.

Second Edition. Crown 8vo., price 3/6 net.
With original "Plea—1900—for the State Registration of Nurses."

**MOCK NURSES OF THE LATEST FASHION.**

By FREDERICK J. GANT, F.R.C.S.,
Consulting Surgeon to the Royal Free Hospital.

BAILLIÈRE, TINDALL & COX,
Henrietta Street, Covent Garden, London.

### NOTICE TO CORRESPONDENTS.

All MSS., Letters, Books for Review, and other matters intended for the Editor should be addressed The Editor, "The Hospital," The Hospital Buildings, 28 & 29 Southampton Street, Strand, W.C.

The Editor cannot undertake to return rejected MS., even when accompanied by stamped directed envelope.

**Notice to Advertisers and Subscribers.**

All Advertisements, Orders for Copies of The Hospital, and Business Communications should be addressed to **THE MANAGER (Not to the Editor),** The Hospital, 28 & 29 Southampton Street, Strand, London, W.C.

The Cost of Subscription, post free, is as follows :—Per week, 3½d.; per quarter, 3s. 9d.; per half-year, 7s. 6d.; per annum, 15s. Foreign and Colonial :—Per annum, 19s. 6d., payable, in all cases, in advance.

**NOTICE.—Vol. XXXIV. of "The Hospital" (April 1903 to September 1903), in handsome cloth binding, is now on sale at the office of this Journal, price 10s. 6d. Cases for binding the Numbers contained in this Volume 2s. Index to Vol. XXXIV., 2½d., post free.**

**The Monthly part for March is now ready. Price 1s., by post, 1s. 3d.**

Mrs. Mary Ann Irwin, who died at Brighton last January, bequeathed £1,000 each to the Home for Incurables, Streatham Common, the Hospital for Sick Children, Great Ormond Street, and the Leicester Infirmary.

### THE STUDENT OF MEDICINE.

### APPOINTMENTS.

**Wallace Ashdowne**, F.R.C.S.Eng., Assistant Surgeon to the Metropolitan Hospital. **J. M. Bennion**, M.B.Cantab., Junior House Surgeon to the Radcliffe Infirmary, Oxford. **Charles Blair**, M.D., F.R.C.S.Eng., Ophthalmic Surgeon to the Royal Hospital, Richmond, Surrey. **D. D. Brown**, M.R.C S., L.R.C.P., Deputy Medical Officer of Health for the Borough of Sunderland. **C. C. Bullmore**, L.R.C.P.&S.Edin., District and Workhouse Medical Officer of the Falmouth Union. **M. L. Clift**, M.R.C.S.Eng., L.S.A., Divisional Surgeon to the City Road Station, "G" Division, Metropolitan Police. **P. D. Cogswell**, M.R.C.S., L.R.C.P., Medical Officer for the Stanton District of the Hinckley Union. **T. H. Davison**, M.D., District Medical Officer of the Hartley Wintney Union. **T. V. De Denne**, M.R.C.S., L.R.C.P., Surgeon to the Victoria Cottage Hospital, Sidmouth. **S. H. Edgelow**, M.R.C.S.Eng., Public Vaccinator for Streaky Bay District, South Australia. **D. Ewart**, M.B., Ch.B., F.R.C.S.Edin., Certifying Factory Surgeon for the Chichester District, Sussex. **C. W. Foster**, M.R.C.S, L.R.C.P., House Surgeon to the Radcliffe Infirmary, Oxford. **A. G. Gibson**, M.B., B Ch Oxon., House Physician to the Radcliffe Infirmary, Oxford. **Herbert James Godwin**, M.B., B.S. Durh., F.R.C.S.Edin., M.R.C.S.Eng., L.R.C.P.Lond., Surgeon Ordinary to the Royal Hants County Hospital, Winchester. **E. H. Harrison**, L.R.C.P.&S.Edin., Medical Officer of the Workhouse of the St. Neots Union. **Randal Herley**, L.R.C P.&S Irel., L.F.P.S.Glasg., Junior House Surgeon to the Clayton Hospital and Wakefield General Dispensary. **Chas. R. Keyser**, F.R.C.S.Eng., Surgical Registrar to the Cancer Hospital. **Charles D. Law**, L.R.C P., L.R.C.S.Edin., Senior Assistant Medical Officer, Crichton Royal Infirmary, Dumfries. **C. W. Lawson**, L.R.C.P.&S.Edin., L.R.C.P.Glasg., District Medical Officer of the Rothbury Union. **F. H. Lawson**, M.R.C.S., L.R.C.P.Lond., District Medical Officer of the Steyning Union. **C. J. Marsh**, L.R.C.P., M.R.C.S., Certifying Factory Surgeon for the Yeovil District, Somerset. **F. Victor Milward**, M.B., F.R.C.S., Medical Referee under the Workmen's Compensation Acts to act for County Court Circuit No. 21 and for the Bromsgrove, Redditch, and Solihull Districts in County Court Circuit No. 22. **W. C. Milward**, M R.C.S., L.R C.P.Lond., District Medical Officer of the Cardiff Union. **W. T. Newton**, M.R.C.S.Eng., L.S.A., Divisional Surgeon to the City Road Station, "G" Division, Metropolitan Police. **W. A. Osborne**, M.D., B.S.R.U.I., Professory of Surgery at the University of Melbourne. **P. H. Rainbird**, L.R C.P.&S Edin., Certifying Factory Surgeon for the Saxilby District, Lincolnshire. **G. E. Richmond**, M.B., B.S., B.A., B.Sc.Lond., D.P.H.Camb., Demonstrator of Hygiene and Public Health at University College, London. **M. Hamblin Smith**, M.R.C.S., L.R C.P., Deputy Medical Officer, H.M. Prison, Manchester, transferred to H.M. Prison, Wandsworth. **Sidney H. Snell**, M.D., B.S.Lond., D.P.H., Assistant Anæsthetist to the

Royal Ear Hospital. Soho. **R. S. Stewart**, M.D.Glasg., C.M. Medical Superintendent of the Glamorgan County Asylum Bridgend. **P. M. Waugh**, M.B., Ch.B.Glasg., Certifying Factory Surgeon for the Rye District, Sussex.

ST. THOMAS'S HOSPITAL.—The following gentlemen have been selected as house officers from Tuesday, March 1st, 1904:—Resident House Physicians: **G. C. Adeney**, M.R.C.S., L.R.C.P.; **E. A. Ross**, M.B., B.C.Cantab.; **R. E. H. Leach**, M.A., M.B., B.Ch. Oxon., M.R.C.S., L.R.C.P. (*Extension*); **G. R. Rickett**, M.A., B.C.Cantab. (*Extension*). House Physicians to Out-patients: **H. C. Lecky**, M.A., M.B., B.Ch.Oxon.; **C. H. Latham**, M.R.C.S., L.R.C.P. Resident House Surgeons: **W. L. Harnett**, M.A., M.B., B.C.Cantab., M.R.C.S., L.R.C.P.; **H. I. Pinches**, M.A., M.B., B.C.Cantab., M.R.C.S., L.R.C.P.; **T. Guthrie**, M.A., M.B., B.C.Cantab., M.R.C.S., L.R.C.P.; **L. E. Wigram**, M.A., M.B., B.C.Cantab. House Surgeons to Out-patients: **H. S. Bennett**, M.R.C.S., L.R.C.P.; **N. C. Carver**, B.A., B.C.Cantab., M.R.C.S., L.R.C.P.; **A. C. Birt**, M.R.C.S., L.R.C.P.; **G. T. Birks**, M.A., M.B., B.C.Cantab. Obstetric House Physicians: (*Senior*) **F. E. Shipway**, M.A., M.B., B.C.Cantab.; (*Junior*) **C. N. Sears**, M.B., B.S.Lond., M.R.C.S., L.R.C.P. Ophthalmic House Surgeon: (*Senior*) **H. D. Hoffman**, B.A., B.C.Cantab. Special Departments: (Throat) **J. C. F. D. Vaughan**, M.R.C.S., L.R.C.P.; (Skin) **B. Higham**, M.R.C.S., L.R.C.P. (*Extension*); **H. Falk**, B.A.Cantab., M.R.C.S., L.R.C.P.

**"The Hospital" Institutional Library.**

"Hospitals and Asylums of the World." 4 Vols., with a Portfolio of Plans, complete £12 12s.

"Burdett's Hospitals and Charities, 1903." 5s. net.

"Burdett's Official Nursing Directory, 1903." 3s. net.

"Cottage Hospitals, General, Fever, and Convalescent." 10s. 6d.

"Uniform System of Accounts for Hospitals and Public Institutions." New Edition. 4s. net.

"Hospital Expenditure: The Commissariat." 2s. 6d.

"A Legal Handbook for the Use of Hospital Authorities." 2s. 6d.

"The Cottage Hospital Case Book or Register of Patients." 10s. and 12s. 6d.

All these are published by the SCIENTIFIC PRESS, Ltd., and may be obtained through any bookseller, or direct from the publishers, 28 and 29 Southampton Street, Strand, London, W.C.

UNIVERSITY COLLEGE, LONDON.

### Useful Handbooks for Nurses.

**THE HUMAN BODY:** Its Personal Hygiene and Practical Physiology. By B. P. Colton.
Profusely Illustrated. Cloth gilt, **5/-** post free.
A clear exposition of the structure and functions of the human body.

**MODERN NURSING IN PRIVATE PRACTICE.** By Sir William Bennett, K.C.V.O., F.R.C.S.
Paper cover **6d.** net, **7d.** post free.

**ART OF MASSAGE.** By Mrs Creighton Hale.

Second Edition, profusely Illustrated. Cloth gilt, **6/-** post free.

**MEDICAL GYMNASTICS,** including the Schott (Nauheim) Movements. A Text-book of Massage and Mechanical Therapeutics. By Axel V. Grafstrom, B.Sc., M.D.
Crown 8vo., Illustrated, cloth, **2/6** post free.

*Complete Catalogue of Nursing Manuals post free on application.*

THE SCIENTIFIC PRESS, LTD., 28 & 29 Southampton Street, Strand, London, W.C.

### Important

**To Midwives,**
**Nurses and**
**Probationers.**

IN THE PRESS. READY SHORTLY.

### A COMPLETE HANDBOOK
OF
### MIDWIFERY.

By J. K. WATSON, M.D., M.B., C.M.,

Author of "A Handbook for Nurses," "Examination of the Urine," &c., &c.

**6/- net**
**6/4**
post free.

This handbook has been designed to meet the requirements of the Central Midwives Board, and is an up-to-date text-book of **all** that the midwife requires to know of the subject treated. The work is produced with the object of rendering it unnecessary for midwives, during their preparation for examination, to consult the larger and more expensive works on the subject written specially for medical men.

**The illustrations are very complete, numbering some 130 separate diagrams, most of which have been specially prepared for this book.**

**6/- net**
**6/4**
post free.

Crown 8vo., about 350 pp., bound in cloth boards.

*N.B.*—As the demand for this work is expected to be very great, Nurses are advised to order the book without delay, as the first edition will in all probability soon be exhausted.

*Of all Booksellers, or of*

THE SCIENTIFIC PRESS, LTD., 28 & 29 Southampton Street, Strand, London, W.C.

**The Best and Most Up to Date.** **2/-** post free.

### PRACTICAL GUIDE TO SURGICAL BANDAGINGS AND DRESSINGS.

By WM. JOHNSON SMITH, F.R.C.S.

Suitable size for the Apron Pocket. Profusely Illustrated. Cloth, 2/- post free.

*Of all Booksellers, or of*

THE SCIENTIFIC PRESS, Ltd., 28 & 29 Southampton Street, Strand. London, W.C.

